# Reframing tricuspid regurgitation in Europe: from marker of risk to therapeutic target

**DOI:** 10.1093/eurheartjsupp/suaf094

**Published:** 2025-12-26

**Authors:** Erwan Donal, Prayuth Rasmeehirun, Marina Petersen Saadi, Emma Bajeux

**Affiliations:** University of Rennes, CHU Rennes, Inserm, LTSI—UMR 1099, Rennes F-35000, France; University of Rennes, CHU Rennes, Inserm, LTSI—UMR 1099, Rennes F-35000, France; University of Rennes, CHU Rennes, Inserm, LTSI—UMR 1099, Rennes F-35000, France; Public Health Department, CHU Rennes, University of Rennes, CIC—UMR 1414, Rennes F-35000, France

**Keywords:** Tricuspid regurgitation, Transcatheter edge-to-edge repair, Cost-effectiveness

## Abstract

Tricuspid regurgitation (TR), once considered a passive marker of advanced cardiac disease, is increasingly recognized as an independent contributor to morbidity, mortality, and healthcare burden. Recent evidence, including the pivotal TRILUMINATE trial and Tri-FR, together with supporting cost-effectiveness models, suggest that the correction of TR with T-TEER systems may improve patient outcomes and offer good value for money in European healthcare settings. This article explores the clinical rationale and economic imperative for considering TR as a modifiable and actionable target in modern cardiology ([Adamo M, Chioncel O, Pagnesi M, Bayes-Genis A, Abdelhamid M, Anker SD *et al.* Epidemiology, pathophysiology, diagnosis and management of chronic right-sided heart failure and tricuspid regurgitation. A clinical consensus statement of the Heart Failure Association (HFA) and the European Association of Percutaneous Cardiovascular Interventions (EAPCI) of the ESC. *Eur J Heart Fail* 2024;**26**:18–33.]).

## Introduction: rethinking the forgotten valve

Tricuspid regurgitation has long been underdiagnosed, undertreated, and underappreciated in clinical cardiology.^1,2^ Historically, its presence was viewed as a secondary phenomenon—an indicator of left-sided heart disease or pulmonary hypertension rather than an independent clinical issue. However, registry studies^3,4,5^ and newer prospective trials have challenged this narrative, revealing that moderate-to-severe TR is independently associated with increased mortality and hospitalization—even when adjusted for left heart disease and pulmonary pressures.

## The burden of disease

TR affects an estimated 1.6 million people in Europe with moderate-to-severe forms, many of whom are elderly and symptomatic. Most patients experience fatigue, peripheral oedema, hepatic congestion, and decreased functional status. In real-world registries, TR severity correlates with poorer outcomes and reduced quality of life—yet less than 10% of patients receive any form of procedural intervention. Even among patients undergoing mitral or aortic surgery, TR is often ignored or worsens post-operatively.^6,7^

Moreover, beyond clinical outcomes, TR is associated with significant economic costs: frequent heart failure hospitalizations, high use of diuretics, and progressive multi-organ dysfunction drive resource use. Given Europe's aging population and rising prevalence of heart failure, TR presents a growing public health challenge.

## Why medical therapy falls short

Guideline-directed medical therapy for TR focuses on symptom relief—typically with loop diuretics and aldosterone antagonists—but does not modify disease progression or valvular pathology. Unlike left-sided valvular disease, there is no pharmacological treatment that reverses TR. As a result, most patients spiral into recurrent decompensations, requiring repeated hospitalizations with declining organ function.

A critical insight from recent trials is that despite adequate medical therapy, patients remain highly symptomatic, and TR progression continues. This therapeutic gap underscores the need for procedural options that directly address the valve mechanism.^8,9,10,11,12,13^

## A paradigm shift: the emergence of transcatheter therapies

Surgical options for isolated TR are limited by high operative risk, with perioperative mortality exceeding 8–10% in many series. The high-risk profile of the typical TR patient—elderly, frail, often with right heart dysfunction and renal or hepatic impairment—renders surgery unattractive.^14,15^

This gap has spurred the development of transcatheter approaches, particularly TEER, specifically engineered for the tricuspid valve. CE-marked in Europe in 2020, one T-TEER offers a catheter-based approach that avoids open-heart surgery, aligning with the risk profile of most TR patients.

## Clinical evidence

### The TRILUMINATE pivotal trial

The TRILUMINATE Pivotal trial, a multicentre randomized controlled study, included 572 patients with severe symptomatic TR. Patients were randomized to either T-TEER + optimal medical therapy or medical therapy alone. At 2-year follow-up, key findings included:

- A 28% relative reduction in annualized heart failure hospitalizations with T-TEER (0.19 vs. 0.26 events/patient-year, *P* = 0.02).
- 84% of T-TEER patients had TR reduced to moderate or less at 2 years vs. 8% in controls.
- Significant and sustained improvements in KCCQ scores (mean +15 points).
- A favourable safety profile, with low rates of procedural complications, stroke, or need for pacemaker implantation.
- In the crossover cohort (control patients switched to T-TEER after 1 year), delayed intervention was still effective, but at the cost of worsening symptoms and higher HF hospitalization rates prior to crossover.^8,10,16^

Importantly, although mortality was not significantly different at 2 years, the divergence in HF hospitalization rates and sustained quality-of-life gains support a therapeutic benefit that may become more evident with longer follow-up. The primary endpoint of TRILUMINATE Pivotal was a hierarchical composite including all-cause mortality or tricuspid valve surgery, heart failure hospitalizations, and quality of life (Kansas City Cardiomyopathy Questionnaire, KCCQ). Among these, the quality-of-life improvement measured by KCCQ contributed most strongly to the primary endpoint result.

## The TRI-FR trial—a European RCT demonstrating clinical benefit of T-TEER

The TRI-FR trial was a landmark investigator-initiated, multicentre, randomized controlled trial conducted in 24 French-Belgium centres. It evaluated the efficacy and safety of T-TEER, in addition to optimal medical therapy, compared with optimal medical therapy alone, in patients with severe symptomatic secondary tricuspid regurgitation (TR) who were deemed at high or prohibitive surgical risk.^13,17^

A total of 300 patients were randomized (151 to T-TEER, 149 to control). Key findings at 12 months included:

- A statistically significant improvement in the primary endpoint, a hierarchical composite of all-cause death, heart failure hospitalization, and change in health status (patient global assessment and NYHA-class).
  - The end-point from Triluminate was also highly in favour of the T-TEER group.
- Reduction in TR severity:
  - At 12 months, 76.7% of patients in the T-TEER group had TR ≤ moderate, compared with only 6.8% in the control group (*P* < 0.001).
- Improvement in health status:
  - The median improvement in KCCQ-OS score at 12 months was +17.9 points in the T-TEER group vs. +3.0 points in the control group (*P* < 0.001).^18^
- Lower heart failure hospitalization burden:
  - Although the individual components of the primary endpoint did not reach significance, the trend towards fewer HF hospitalizations and better quality of life favoured the intervention group.
- Safety:
  - T-TEER was associated with a low rate of serious device-related adverse events (2.3% at 30 days), and no significant excess in mortality or stroke compared to controls.
- Mechanistic insights:
  - Echocardiographic assessment showed the relevance for getting the best possible reduction in TR. There has been clearly demonstrated the relationship between the quality of the TR correction and the change in the quality of life. It could be extrapolated to a beneficial effect on the late occurrence of hospitalization and death.

The TRI-FR trial is the first positive randomized controlled trial conducted in Europe to demonstrate that treating severe TR with transcatheter therapy improves clinical outcomes, functional status, and echocardiographic parameters compared with medical therapy alone. Its success reinforces the role of T-TEER as a viable and effective treatment strategy in selected high-risk patients, particularly those with atrial functional TR, preserved right ventricular function, and suitable valve anatomy.

## Medico-economic validation: cost-effectiveness of T-TEER in France

The current official cost of the TriClip™ procedure in France (€21 000–€22 000 for the device, excluding procedural and hospitalization costs). The exact values vary across European countries but fall within a similar range.

Using a Markov model from the French public payer perspective, a cost-effectiveness study modelled T-TEER over a 10-year horizon in patients mirroring those in the TRILUMINATE trial.^10^ Key outcomes included:

- +1.18 life-years gained and +1.38 QALYs vs. medical therapy.
- Incremental cost-effectiveness ratio (ICER): €22 005/QALY—well below commonly accepted thresholds.
- Robust sensitivity analyses confirmed the model's stability under various assumptions, including real-world mortality inputs (bRIGHT registry.^19^) The b-RIGHT registry is highly reassuring about the robustness of the therapy with stable results recently demonstrated at 2-years follow-up.
- Costs were driven primarily by device acquisition and procedure but offset by reduced hospitalization and improved utilities.

**Figure 1:**
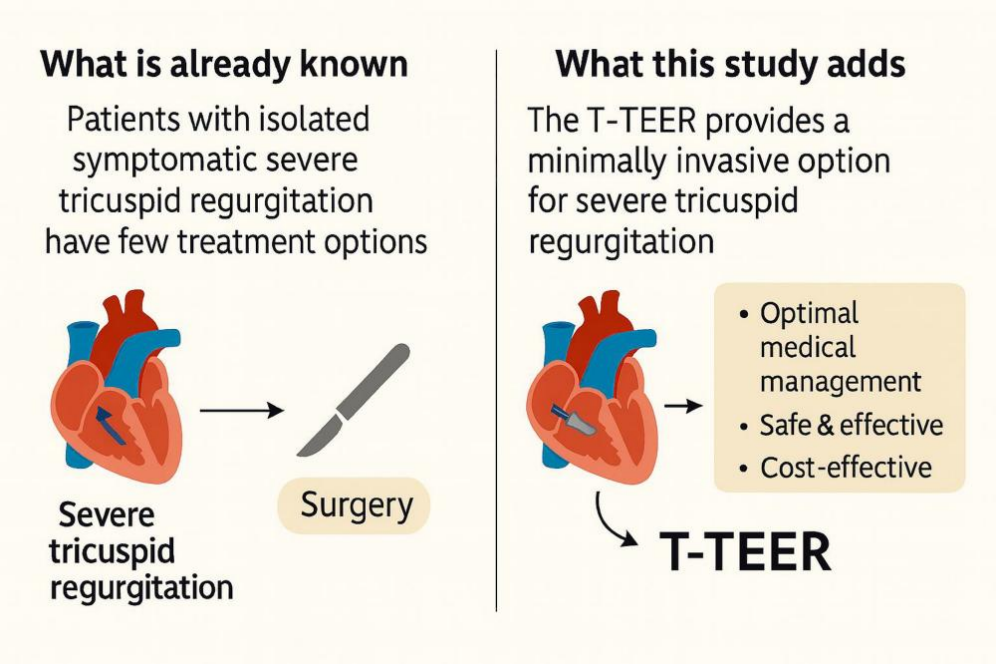
Schematic representation of the unmet need and the value to consider tricuspid transcatheter edge-to-edge repair (T-TEER).

**Figure 2:**
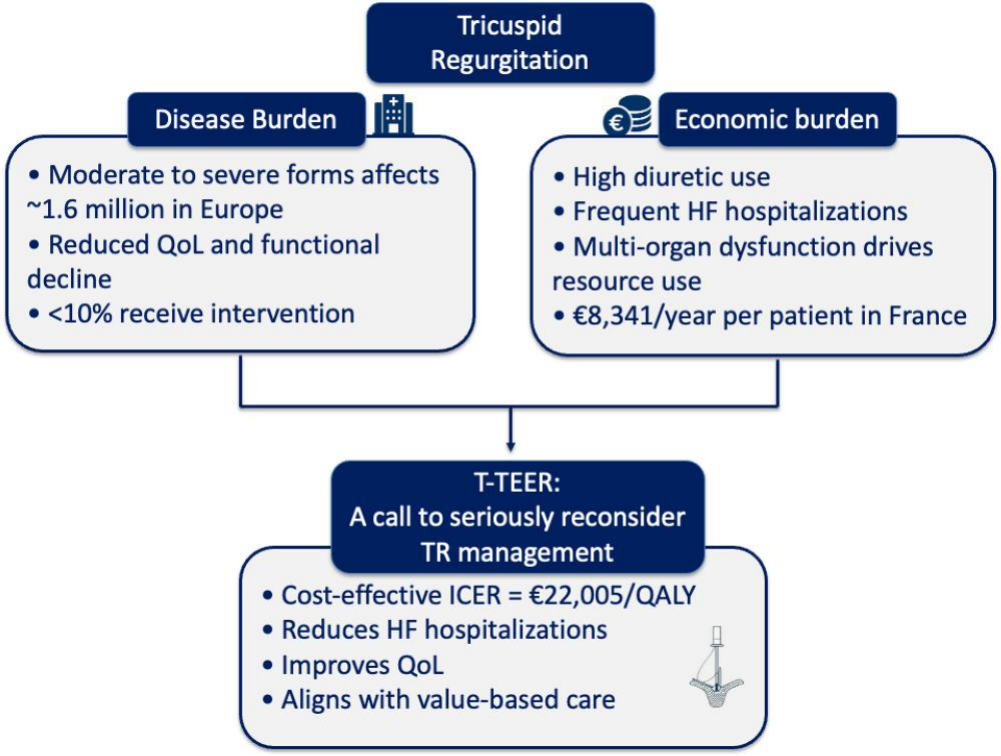
Summary representation of the main value of transcatheter correction of tricuspid regurgitations.

These findings demonstrate that T-TEER is not only clinically beneficial but economically viable in the French system—and potentially across similar European health systems with shared reimbursement structures (*Figures 1* and *2*).

## Beyond France: implications for European health policy

While health technology assessment (HTA) thresholds and reimbursement mechanisms differ between European countries, the economic value demonstrated by the French cost-effectiveness model for T-TEER is broadly applicable across Europe for several reasons:

- Comparable healthcare structures and payer expectations:
  France's public payer model shares key characteristics with many EU systems (e.g. Italy, Germany, Spain), where interventions are evaluated on QALY-based thresholds and hospital cost offsets from reduced rehospitalizations. The T-TEER ICER in France—€22 005/QALY—falls well below conventional willingness-to-pay thresholds of €30 000–€50 000 in most EU settings.
- Cross-national modelling confirms value:

Similar Markov modelling of T-TEER has been conducted in:

- Poland and Turkey, with ICERs between €20 000–€24 000/QALY, indicating consistency in cost-effectiveness under differing unit costs and healthcare resource utilizations.
- UK, where NICE considers interventions cost-effective below £30 000/QALY. While no formal NICE appraisal exists yet for T-TEER, a probabilistic sensitivity analysis using UK-specific costs and utilities confirmed that the intervention would likely meet NICE cost-effectiveness criteria^20^

- Germany and DRG-based systems:
  In Germany and Austria, reimbursement is governed by Diagnosis Related Groups (DRGs), which are highly sensitive to reductions in hospital admissions. As T-TEER reduces heart failure hospitalizations (TRILUMINATE) and improves KCCQ scores, it is likely to deliver net cost savings in elderly frail populations—particularly when early rehospitalizations are costly. In TRILUMINATE, the hierarchical composite endpoint significantly favoured the T-TEER group, driven primarily by improvements in health status (KCCQ) and reduced heart failure hospitalizations, while mortality remained similar between groups at 2-year.
- European Society for Cardiology and EUnetHTA alignment (https://www.eunethta.eu/hta-core-model):
  The EUnetHTA Core Model, adopted for multi-country evaluations, promotes transferability of economic models when patient populations, disease progression, and care pathways are similar—which is the case for TR patients in Europe. The ESC Heart Failure guidelines also emphasize reducing hospitalization burden in guideline-directed management, underscoring the clinical-economic alignment of interventions like T-TEER.^21^

The cost-effectiveness of T-TEER observed in the French model is not unique to France. Given shared patient profiles, disease burden, and healthcare goals across EU nations, the economic rationale for adopting T-TEER is sound and transferable. National HTA bodies should consider this evidence when evaluating reimbursement pathways.

## Unmet needs and future directions

Despite encouraging data, several barriers remain:

- Lack of awareness or reluctance to refer patients for TR evaluation and treatment.
- Limited access to specialized structural heart teams and centres with TEER capability.
- Reimbursement and regulatory hurdles in some EU countries.
- A need for longer-term data (>5 years).

Future priorities include:

- Developing risk scores to identify patients who benefit most.
- Integrating TR screening into heart failure pathways.
- Expanding TEER training and programme capacity.
- Evaluating combinations with other transcatheter therapies. Results have been presented for transcatheter tricuspid valve replacement -TTVR) and the relative value of this new therapy as compare to T-TEER remains completely unknown.

## Conclusion: a call for action

It is time to reframe tricuspid regurgitation—not as a passive marker of disease severity but as a modifiable driver of patient outcomes. With the availability of transcatheter therapies like T-TEER, supported by randomized data and cost-effectiveness models, European cardiology has both the tools and the evidence to act.

If health systems embrace this opportunity, they can alleviate the burden of heart failure hospitalizations, improve patient quality of life, and invest resources in interventions that deliver long-term value. The forgotten valve no longer needs to be forgotten.

## Data Availability

This is a review document, no specific new data are provided.

## References

[1] Adamo M, Chioncel O, Pagnesi M, Bayes-Genis A, Abdelhamid M, Anker SD et al Epidemiology, pathophysiology, diagnosis and management of chronic right-sided heart failure and tricuspid regurgitation. A clinical consensus statement of the Heart Failure Association (HFA) and the European Association of Percutaneous Cardiovascular Interventions (EAPCI) of the ESC. Eur J Heart Fail 2024;26:18–33. 38131233 10.1002/ejhf.3106

[2] Guerin A, Dreyfus J, Tourneau L, Sportouch T, Lairez C, Eicher O et al Secondary tricuspid regurgitation: Do we understand what we would like to treat? Arch Cardiovasc Dis 2019;112:642–51. 31351805 10.1016/j.acvd.2019.04.010

[3] Topilsky Y, Maltais S, Medina Inojosa J, Oguz D, Michelena H, Maalouf J et al Burden of Tricuspid Regurgitation in Patients Diagnosed in the Community Setting. JACC Cardiovasc Imaging 2019;12:433–42. 30121261 10.1016/j.jcmg.2018.06.014

[4] Topilsky Y, Nkomo VT, Vatury O, Michelena HI, Letourneau T, Suri RM et al Clinical outcome of isolated tricuspid regurgitation. JACC Cardiovasc Imaging 2014;7:1185–94. 25440592 10.1016/j.jcmg.2014.07.018

[5] Messika-Zeitoun D, Verta P, Gregson J, Pocock SJ, Boero I, Feldman TE et al Impact of tricuspid regurgitation on survival in patients with heart failure: a large electronic health record patient-level database analysis. Eur J Heart Fail 2020;22:1803–13. 32367642 10.1002/ejhf.1830

[6] Enriquez-Sarano M, Messika-Zeitoun D, Topilsky Y, Tribouilloy C, Benfari G, Michelena H. Tricuspid regurgitation is a public health crisis. Prog Cardiovasc Dis 2019;62:447–51. 31707061 10.1016/j.pcad.2019.10.009

[7] Cahill TJ, Prothero A, Wilson J, Kennedy A, Brubert J, Masters M et al Community prevalence, mechanisms and outcome of mitral or tricuspid regurgitation. Heart 2021;107:1003–9. 33674352 10.1136/heartjnl-2020-318482

[8] Kar S, Makkar RR, Whisenant BK, Hamid N, Naik H, Tadros P et al Two-year outcomes of transcatheter edge-to-edge repair for severe tricuspid regurgitation: The TRILUMINATE pivotal randomized trial. Circulation 2025;151:1630–8. 40159089 10.1161/CIRCULATIONAHA.125.074536

[9] Lurz P, Stephan von Bardeleben R, Weber M, Sitges M, Sorajja P, Hausleiter J et al Transcatheter edge-to-edge repair for treatment of tricuspid regurgitation. J Am Coll Cardiol 2021;77:229–39. 33478646 10.1016/j.jacc.2020.11.038

[10] Sorajja P, Whisenant B, Hamid N, Naik H, Makkar R, Tadros P et al Transcatheter Repair for Patients with Tricuspid Regurgitation. N Engl J Med 2023;388:1833–42. 36876753 10.1056/NEJMoa2300525

[11] Donal E, Galli E, Bidaut A. Advocacy for more consideration of the secondary tricuspid regurgitation. Heart 2019;105:1221–2. 31142590 10.1136/heartjnl-2019-315262

[12] Donal E, Leurent G, Iung B. Are we right to believe in the value of transcatheter treatment of secondary tricuspid regurgitation? J Am Coll Cardiol 2021;77:240–2. 33478647 10.1016/j.jacc.2020.11.037

[13] Donal E, Dreyfus J, Leurent G, Coisne A, Leroux PY, Ganivet A et al Transcatheter edge-to-edge repair for severe isolated tricuspid regurgitation: The Tri.Fr randomized clinical trial. JAMA 2025;333:124–32. 39602173 10.1001/jama.2024.21189 PMC11733701

[14] Dreyfus J, Flagiello M, Bazire B, Eggenspieler F, Viau F, Riant E et al Isolated tricuspid valve surgery: impact of aetiology and clinical presentation on outcomes. Eur Heart J 2020;41:4304–17. 32974668 10.1093/eurheartj/ehaa643

[15] Dreyfus J, Galloo X, Taramasso M, Heitzinger G, Benfari G, Kresoja KP et al TRI-SCORE and benefit of intervention in patients with severe tricuspid regurgitation. Eur Heart J 2023;45:586–97. 10.1093/eurheartj/ehad585 37624856

[16] Arnold SV, Goates S, Sorajja P, Adams DH, von Bardeleben RS, Kapadia SR et al Health status after transcatheter tricuspid-valve repair in patients with severe tricuspid regurgitation. J Am Coll Cardiol 2024;83:1–13. 37898329 10.1016/j.jacc.2023.10.008

[17] Donal E, Leurent G, Ganivet A, Lurz P, Coisne A, De Groote P et al Multicentric randomized evaluation of a tricuspid valve percutaneous repair system (clip for the tricuspid valve) in the treatment of severe secondary tricuspid regurgitation Tri.Fr Design paper. Eur Heart J Cardiovasc Imaging 2021;23:1617–27. 10.1093/ehjci/jeab255 34871375

[18] Istratoaie S, de Groote P, Karam N, Trochu JN, Leurent G, Coisne A et al Quality of life after transcatheter tricuspid valve repair: results from the Tri.FR trial. ESC Heart Fail 2025;12:3053–61. 40387042 10.1002/ehf2.15327 PMC12287848

[19] Lurz P, Rommel KP, Schmitz T, Bekeredjian R, Nickenig G, Mollmann H et al Real-world 1-Year Results of Tricuspid Edge-to-Edge Repair from the bRIGHT Study. J Am Coll Cardiol 2024;84:607–16. 38759905 10.1016/j.jacc.2024.05.006

[20] Maisano F, Hahn R, Sorajja P, Praz F, Lurz P. Transcatheter treatment of the tricuspid valve: current status and perspectives. Eur Heart J 2024;45:876–94. 38426859 10.1093/eurheartj/ehae082

[21] McDonagh TA, Metra M, Adamo M, Gardner RS, Baumbach A, Bohm M et al 2021 ESC Guidelines for the diagnosis and treatment of acute and chronic heart failure. Eur Heart J 2021;42:3599–726. 34447992 10.1093/eurheartj/ehab368

